# Changes in Telomere Length in Leukocytes and Leukemic Cells after Ultrashort Electron Beam Radiation

**DOI:** 10.3390/ijms25126709

**Published:** 2024-06-18

**Authors:** Tigran Harutyunyan, Anzhela Sargsyan, Lily Kalashyan, Hovhannes Igityan, Bagrat Grigoryan, Hakob Davtyan, Rouben Aroutiounian, Thomas Liehr, Galina Hovhannisyan

**Affiliations:** 1 Laboratory of General and Molecular Genetics, Research Institute of Biology, Yerevan State University, Alex Manoogian 1, Yerevan 0025, Armenia; tigranharutyunyan@ysu.am (T.H.); angela.sargsyan@ysu.am (A.S.); lilikalashyan@ysu.am (L.K.); hovhannes.igityan@ysu.am (H.I.); genetik@ysu.am (R.A.); galinahovhannisyan@ysu.am (G.H.); 2 Department of Genetics and Cytology, Yerevan State University, Alex Manoogian 1, Yerevan 0025, Armenia; 3 CANDLE Synchrotron Research Institute, Acharyan 31, Yerevan 0040, Armenia; grigory@asls.candle.am (B.G.); davtyan@asls.candle.am (H.D.); 4 Institute of Human Genetics, Jena University Hospital, Friedrich Schiller University, Am Klinikum 1, D-07747 Jena, Germany

**Keywords:** laser-generated electron beams, ionizing radiation, telomere length, Q-FISH, human blood leukocytes, K562 cell line

## Abstract

Application of laser-generated electron beams in radiotherapy is a recent development. Accordingly, mechanisms of biological response to radiation damage need to be investigated. In this study, telomere length (TL) as endpoint of genetic damage was analyzed in human blood cells (leukocytes) and K562 leukemic cells irradiated with laser-generated ultrashort electron beam. Metaphases and interphases were analyzed in quantitative fluorescence in situ hybridization (Q-FISH) to assess TL. TLs were shortened compared to non-irradiated controls in both settings (metaphase and interphase) after irradiation with 0.5, 1.5, and 3.0 Gy in blood leukocytes. Radiation also caused a significant TL shortening detectable in the interphase of K562 cells. Overall, a negative correlation between TL and radiation doses was observed in normal and leukemic cells in a dose-dependent manner. K562 cells were more sensitive than normal blood cells to increasing doses of ultrashort electron beam radiation. As telomere shortening leads to genome instability and cell death, the results obtained confirm the suitability of this biomarker for assessing genotoxic effects of accelerated electrons for their further use in radiation therapy. Observed differences in TL shortening between normal and K562 cells provide an opportunity for further development of optimal radiation parameters to reduce side effects in normal cells during radiotherapy.

## 1. Introduction

Laser-generated particles are a promising option for radiotherapy due to combining a compact, cost-efficient treatment unit with the physical advantages of charged particle beams [1,2]. As noted by Rigaud et al. [3], in conventional cancer therapy, under low-dose rates the time necessary for energy deposition is very long compared with the dynamics of early molecular and cellular responses. Laser-generated electron bunches demonstrate ultrashort beam pulses in femto- to picosecond range, which ensures rapid delivery of doses to cells, high peak dose rate, monoenergetic spectral profile, and little lateral spread. The resulting more accurate targeting of cells allows for minimal impact on surrounding tissue, while preserving the antitumor effect [4,5,6]. Laser-generated particles’ acceleration technologies have advanced significantly over the past decade, leading to impressive improvements in beam quality, stability, and reproducibility of parameters [7,8,9]. Advances in the development of laser-generated particle acceleration technology have enabled serious consideration of its medical application in cancer radiotherapy.

Before entering the medical practice, the radiobiological effects of laser-generated electrons have to be established. To date, only few studies evaluated the genetic effects of laser-generated electron beams using various endpoints. Laser-accelerated electrons were shown to increase DNA damage as assessed by the comet assay in human skin carcinoma cells [3]. Irradiation of human lymphocytes, fibroblasts, and Chinese Hamster Ovary (CHO) cells with laser-accelerated electrons increased levels of DNA double-strand breaks, similar to the ones induced by conventional electron beams, as substantiated by γ-H2AX assay [10]. No considerable differences were found in the effects of laser-accelerated and conventional electron beams on normal tissue cells concerning the number of double-stranded DNA breaks [11]. Harutyunyan et al. [12] showed that irradiation with laser-generated electrons can induce DNA copy number variations in human blood leukocytes in vitro. DNA damage evaluated by comet assay in K562 (human chronic myeloid leukemia) cells irradiated with laser-generated electron beams revealed a shift towards higher amounts of damage and a broadening in distribution with a consequent decrease in their reparability compared with non-irradiated cells [13]. Electron beam radiation compared to X-rays is characterized by significantly slower γ-H2AX foci repair and strong apoptosis induction, accompanied by a slight increase in micronuclei formation in MRC5 (human fetal lung fibroblasts) cells [14]. Delayed repair of γH2AX and 53BP1 foci (markers of DSBs) was also found after exposure of HeLa (human cervical cancer cells) and A549 (human lung carcinoma) cells to laser-generated ultrashort pulsed electron irradiation compared to conventional quasi-continuous electron irradiation [15]. Thus, according to Babayan et al. [13,14,15], laser-generated electrons increase the formation of more complex and difficult-to-repair DNA damage than conventional radiation. Laser-accelerated electrons irradiation study in vivo showed an increase of DNA damage in the spleen, thymus, and bone marrow of whole-body irradiated Wistar rats [16]. The only available study of the effects of laser-accelerated electrons using telomeres as an endpoint showed that accelerated electrons were more effective in telomere shortening compared to X-rays, and also in inducing micronuclei in human peripheral blood lymphocytes (PBL) [17]. 

Telomere length (TL) can be measured by various methods, including quantitative fluorescence in situ hybridization (Q-FISH) applying peptide nucleic acid (PNA) telomere oligonucleotide probe and appropriate digital image software (freely available at https://demarzolab.pathology.jhmi.edu/telometer/ (accessed on 15 January 2023)) for capture and quantification of fluorescence signals [18]. PNA probes are highly resistant to chemical and enzymatic degradation and provide higher signal intensity than DNA probes. Each PNA probe recognizes three telomeric repeats (T_2_AG_3_), so the intensity of the fluorescent signal from telomeric PNA probes that hybridize to a given telomere is directly proportional TL [19]. Q-FISH enables the analysis of TL in individual (single) cells compared to genomic DNA-based methods that estimate TL in DNA isolated from a population of cells [19]. 

By Q-FISH on metaphases TL quantification of individual chromosome ends at the single-cell level are feasible. However, the requirement for metaphase chromosomes restricts analysis to actively proliferating cells. Q-FISH on interphase nuclei permits to overcome this limitation. Q-FISH on interphase nuclei allows the measurement of aggregations of few telomeres also called “telomere spots” instead of the telomeres at the ends of individual chromosomes [19,20]. According to Montpetit et al. [20], interphase and metaphase versions of Q-FISH can access TL in single cells with the resolution of 0.15–0.3 kb. Other authors give an average resolution of 0.2 kb [21,22].

The Q-FISH-based approach permits the detection of not only the mean TL, but also the intercellular heterogeneity of telomere signals [23,24] as well as the determination of the fraction of shortest telomeres. It is the shortened telomeres that can activate the DNA damage response, leading to cell cycle arrest and senescence. Therefore, the fraction of shortest telomeres is a more effective endpoint of radiation effect than average TL [19,25,26,27].

Increasing interest has recently been focused on the biological effects of laser-generated electron beams for their clinical applications. The genotoxic impact of laser-accelerated electrons has been investigated using different DNA damage endpoints, whereas other important aspects, such as telomere dysfunction, have been less studied. Only one publication reports on the effect of laser-driven electron pulse irradiation on telomeres [17]. The understanding of the biological effectiveness of laser-generated electron beams can be extended by introducing additional radiobiological endpoints. The purpose of this study was to evaluate TL detected by Q-FISH as a biomarker of laser-generated electron beam irradiation. At first, the ability of interphase and metaphase Q-FISH to assess changes in TL in irradiated normal human blood cells was studied; in a second step, interphase Q-FISH version was applied on K562 leukemic cells. Performing Q-FISH in interphase nuclei allows for measurements in nucleated cells of numerous tissues regardless of cell division; this approach can be further used as one of the endpoints of the genotoxic effects of accelerated electrons. A second focus of the study was to compare the sensitivity of normal and tumor cells to laser-generated electron beam irradiation accessed by TL alterations.

## 2. Results

The effects of laser-generated electrons on TL were studied in human normal blood leukocytes and human chronic myeloid leukemia K562 cells using the Q-FISH method. 

Radiation doses which do not cause a pronounced cytotoxic effect were selected based on previous in vitro studies in blood [12] and K562 cells [13]. 

### 2.1. TL in Interphase Nuclei and Metaphase Chromosomes of Blood Cells Irradiated with Laser-Generated Electrons

**Figure 1:**
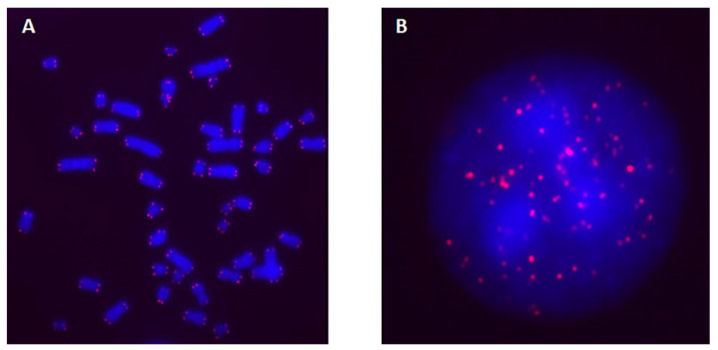
Representative images of telomeres in metaphase chromosomes (**A**) and interphase nuclei (**B**) of blood leukocytes are shown by a Cy3-labeled PNA probe (red) with DAPI counterstaining (blue). The images were captured at 100× magnification using CoolCube1 camera on a Zeiss fluorescent microscope (GmbH, Altlussheim, Germany).

Q-FISH-signals reflecting TL were measured on metaphase chromosomes and interphase nuclei in blood cells of four donors. Figure 1 shows telomeres on metaphase chromosomes (Figure 1A) and interphase nuclei (Figure 1B) that were hybridized with a Cyanine3 (Cy3)-labeled PNA probe and DAPI (4′,6-diamidino-2-phenylindole) counterstaining.

No statistically significant difference in TL was found between four donors (Table 1). Thus, we combined the data obtained in groups according to radiation doses.

**Figure 2:**
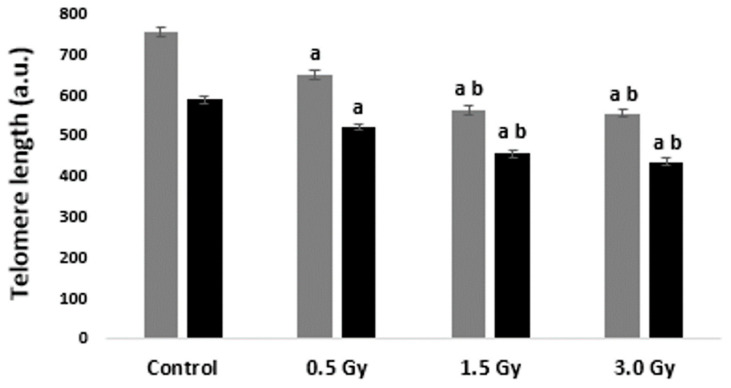
Mean TLs ± SE (a.u.—arbitrary units) in normal blood leukocytes, irradiated with the laser-generated electron beam, analyzed by metaphase (gray bars) and interphase (black bars) Q-FISH. Samples of each donor were analyzed in triplicates. ^a^ *p* < 0.05—statistically significant difference compared with control, ^b^ *p* < 0.05—statistically significant difference compared with 0.5 Gy.

Statistical analysis showed TL shortening in both metaphase chromosomes and interphase nuclei after irradiation with doses of 0.5, 1.5, and 3.0 Gy (*p* < 0.05) (Figure 2).

According to the data obtained both (metaphase and interphase) versions of Q-FISH were able to detect the same dose-dependent telomere shortening effects upon irradiation. TLs in irradiated cells were significantly different from the unirradiated control. The effects of the studied doses also differed significantly from each other, with the exception of doses of 1.5 and 3.0 Gy. All together, these results validate the same sensitivity of metaphase and interphase Q-FISH for the determination of TL changes in cells irradiated with a laser-generated electron beam.

### 2.2. TL in Interphase Nuclei of K562 Cells Irradiated with Laser-Generated Electrons

**Figure 3:**
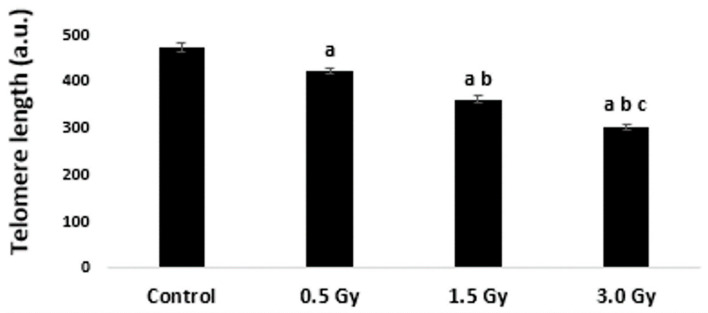
Mean TLs ± SE (a.u—arbitrary units) in interphase nuclei of K562 cells irradiated with the laser-generated electron beam. Pooled data from four independent experiments in 120–160 cells per treatment are shown. ^a^ *p* < 0.05—statistically significant difference compared with control, ^b^ *p* < 0.05—statistically significant difference compared with 0.5 Gy, ^c^ *p* < 0.05—statistically significant difference compared with 1.5 Gy.

The same efficiency of the metaphase and interphase Q-FISH in blood leukocytes was sufficient reason to select the interphase version of Q-FISH to evaluate the effects of irradiation on TL in K562 cells. To obtain results comparable to the blood cells of four donors, each experimental variant in K562 cells was conducted in quadruplicate, and in total, 120–160 interphases were analyzed in control and for each radiation dose. Significant shortening of TLs was found in irradiated K562 cells (Figure 3). According to the results obtained, the telomeres in interphases of non-irradiated K562 cells are shorter than the telomeres of normal blood leukocytes (470.75 a.u. versus 586.54 a.u.) and the same difference is preserved in irradiated cells.

### 2.3. Correlation between TL and Doses in Blood and K562 Cells

**Figure 4:**
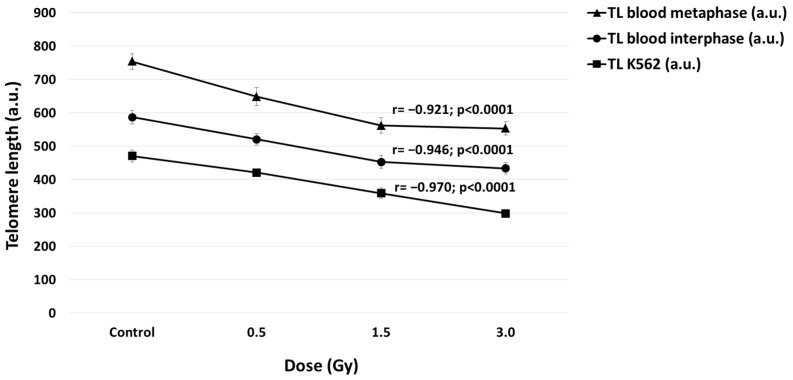
Correlations between TLs (a.u.—arbitrary units) and doses of laser-generated electron beam in metaphases and interphases of normal blood leukocytes and interphases of K562 cells. TLs in metaphases (r = −0.921; *p* < 0.0001) and interphases (r = −0.946; *p* < 0.001) of normal blood leukocytes and interphases of K562 cells (r = −0.970; *p* < 0.0001) were inversely correlated with irradiation doses.

Spearman’s rank correlation analysis indicates dose-dependent telomere shortening both in metaphase chromosomes (r = −0.921; *p* < 0.0001) and interphase nuclei (r = −0.946; *p* < 0.001) of blood leukocytes and in interphase nuclei of K562 cells (r = −0.970; *p* < 0.0001) (Figure 4).

### 2.4. Distribution of TLs in Blood and K562 Cells

In addition to the average TL, cell-to-cell distribution of TLs and the frequency of cells with the shortest telomeres (<20th percentile) was also analyzed. In our study, short telomeres were considered as a TL <20th percentile of the corresponding control, as was previously reported [28,29,30]. 

**Figure 5:**
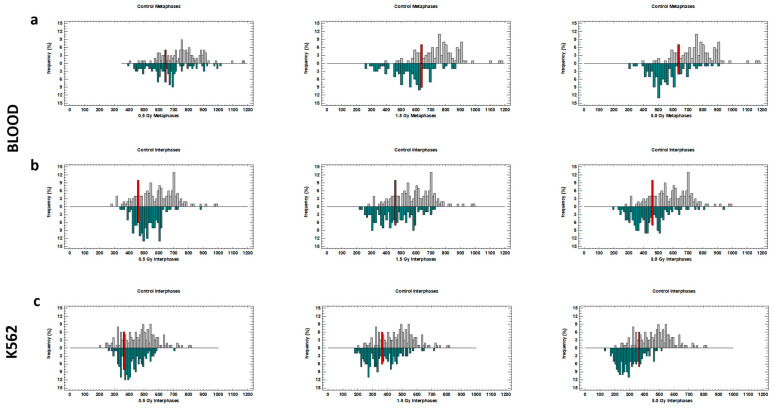
Distributions of TLs in metaphase chromosomes (**a**) and interphase nuclei (**b**) of blood leukocytes and interphase nuclei (**c**) of K562 cells irradiated with the laser-generated electron beam. Pooled data from four independent experiments in 120–160 cells per treatment are shown. The gray and blue bars indicate the frequencies of telomeres in control and irradiated variants, respectively. The red bar indicates the 20th percentile of the corresponding control.

Figure 5 shows the distribution of TLs, and the red bars indicate the 20th percentile of the TL of the corresponding control. A Kolmogorov–Smirnov test revealed statistically significant differences between the distributions of TLs in irradiated and control blood and K562 cells. Significant differences between irradiation doses (with the exception of doses 1.5 and 3.0 Gy in metaphase chromosomes and interphase nuclei of blood leukocytes) were also revealed. The differences obtained coincide with the results of comparing the mean TL values using the Kruskal–Wallis test and confirm radiation-induced telomere shortening. Thus, an increase in the proportion of the shortest telomeres with increasing radiation doses in all experimental variants was detected (Table 2).

## 3. Discussion

The development of radiotherapy must continue to improve ways of killing cancer cells with fewer side effects on nearby healthy cells. The interest in assessing the biological effects of laser-generated particle beams is motivated by their differences from conventional radiation generated by classical accelerators or sources, which can be used to develop new radiotherapy strategies minimizing the impact on surrounding tissues [6]. 

Our study examined the effects of laser-generated electron beams on the telomeres of both normal blood cells and human chronic myeloid leukemia K562 cells of the same tissue origin to compare potential therapeutic and side effects.

Primary cells from patients with chronic myeloid leukemia (CML) as well as CML-derived cell lines (including K562) are known as suitable models for studying telomeres and telomerase activity, mainly due to easy access to primary tumor cells and the availability of control tissue [31,32,33]. Furthermore, K562 cells were used before for testing new therapeutic approaches for CML, which is particularly important in cases of imatinib-mesylate (Gleevec) resistance [34,35]. This is in line with the aim of our study to evaluate the potential radiotherapeutic effect of accelerated electrons. 

Given the specificity of exposure to accelerated electrons as opposed to conventional ionizing radiation [6], our study provided a preliminary assessment of the comparability of metaphase and interphase TL measurements in blood cells. According to our results, telomeres in metaphases were longer than in interphases of control and irradiated blood leukocytes. We hypothesize that cells with a higher level of proliferative activity and, accordingly, longer telomeres are predominantly found at the metaphase stage. Cells with less potential to divide may be more likely to enter a quiescent state. Thus, shorter telomeres predominate in interphase nuclei. Another reason could be differences in visualization of metaphase and interphase telomeres using Q-FISH. Metaphase images allow more accurate analysis due to better signal spread and, accordingly, visualization of signals. Canela et al. [23] showed that the average metaphases TL of 11 human and mice cell lines can be both longer and shorter than in interphases (however, no statistical analysis or explanation of this difference is presented).

Despite differences in absolute TL, similar dose-dependent telomere shortening was obtained in metaphase and interphase of blood cells, allowing the application of a cell division-free, less labor-intensive and time-consuming interphase Q-FISH on K562 cells. The simultaneous use of the metaphase and interphase telomere Q-FISH in the panel of human and mouse cell lines [23], in T-lymphocytes of individuals with Down syndrome, dementia, and mild cognitive impairment [36,37] and in bone marrow cells from patients with myelodysplastic syndrome [38] also showed the high sensitivity and comparability of both approaches.

In control cells, TLs were shorter in interphase nuclei of K562 cells compared to normal blood leukocytes. This is consistent with the literature that TL in tumors is usually (but not always) shorter than in corresponding normal tissue because of their extensive replicative history without sufficient telomerase to maintain TL [39,40]. These results are also in line with the data showing shorter telomeres in patients with CML than in healthy individuals [41,42]. Keller et al. [31] have estimated, based on the 1 kb difference of average TL between normal and chronic phase CML cells, that malignant cells may undergo approximately 10 times more cell divisions than normal cells. 

According to our results, dose-dependent telomere shortening was revealed in normal blood metaphases and interphases as well as in interphases of K562 cells irradiated with laser-generated electron beams. Significant differences in TLs were found between all radiation doses in K562 cells, while in normal blood cells, the effects of doses of 1.5 and 3.0 Gy did not differ, which indicates a higher sensitivity of leukemic cells to radiation. 

In addition to average TL, Q-FISH data were used to analyze intercellular TL distributions and proportion of cells with the shortest telomeres (shorter than the 20th percentile of the control variant). The distributions of TLs of K562 cells significantly differ at all irradiation doses. In contrast, the distributions of blood cells at doses of 1.5 and 3.0 Gy are the same, which coincides with the results of comparison of medians. Shifts in the distribution of TLs revealed an increase in the number of shortest telomeres after irradiation of blood cells and K562 cells, with the most pronounced difference between them at a dose of 3.0 Gy. Metaphase Q-FISH shows higher levels of the shortest telomeres than interphase Q-FISH in blood cells, probably due to the higher proliferative activity of cells at the metaphase stage. In addition, the overlaps of telomeric parts of chromosomes in interphase nuclei might also be involved in the observed results. 

Our results on telomere shortening in irradiated cells are consistent with literature data on the ability of radiation to damage DNA and in particular the telomeres. Ionizing radiation can damage DNA directly by energy transfer from particles to matter, or indirectly by forming a pool of free radicals after the radiolysis of water molecules [6,43,44]. Radiation-induced increases in oxidative stress are known to accelerate telomere shortening and dysfunction [45,46], which can be reduced by antioxidants [47]. Telomeres are critically susceptible to oxidative damage due to their guanine-rich repetitive sequences, which are known as preferred sites for the production of the 8-oxoguanine [46,48,49,50]. 8-oxoguanine terminates telomere elongation due to replication fork arrest at telomeres [51]. Moreover, radiation-induced DNA breaks at telomeres are repaired less efficiently than in other sequences likely due to the T-loop structure reducing access to DNA repair machinery [48]. Miller et al. [52] suggested that the non-homologous end joining (NHEJ) repair deficiency in regions near telomeres may result from the activity of telomere proteins in preventing chromosome fusion. DNA damage foci induced by X-ray, H_2_O_2_, and neocarzinostatin in vitro in cultured human embryonic fibroblasts, MRC5, and mouse embryonic fibroblasts were shown to be preferentially located at telomeres. Moreover, telomere-associated foci are longer lived than non-telomeric foci [53]. Large fraction of ionizing radiation-induced persistent DNA damage response (DDR) markers were shown to be associated with telomeric DNA in cultured cells BJ, MRC5, and HeLa as well as in neurons of irradiated adult mice [54]. While the number of DDR foci per cell progressively declines, the fraction of those that co-localize with a telomeric signal gradually increases. Fumagalli et al. [54] proposed that linear genomes are not uniformly reparable and that telomeric DNA cannot repair exogenous DNA damage in full. Accelerated telomere shortening and dysfunction associated with oxidative stress in numerous human studies, mouse models, and cell cultures were summarized in review of Barnes et al. [46]. Armstrong and Boonekamp [55] provided support for the effect of oxidative stress on telomere attrition in humans and other vertebrate species in vivo using meta-analysis.

Reports of changes in TL following ionizing radiation exposure are contradictory, suggesting both lengthening and shortening depending on the type and conditions of irradiation. Accelerated telomere shortening and decreased telomerase activity have been shown in γ-irradiated normal human T-lymphocytes and fibroblasts [56], as well as in panels of mammary epithelial and hematopoietic cell lines exposed to γ-rays or high-energy particles [57]. In contrast, low-energy protons have been shown to induce a significant increase in TL in human primary fibroblasts and mouse embryonic fibroblasts [58,59]. This phenomenon has not been completely explained; the authors speculated that telomere elongation may be the result of repair of chromosome damage by acquiring telomeric sequences [58].

We found the only study that analyzed the length of telomeres in human peripheral blood lymphocytes irradiated with laser-driven ultrashort electron beams [17]. Mean TL was found to be reduced in a significant dose-dependent manner after irradiation with both laser-driven electron beam and conventional X-ray radiation. Laser-driven electron beams were found to be more effective in telomere shortening compared to X-rays, confirming the specificity of the effects of accelerated electrons and the need for further study of their biological effects. 

Data from in vivo studies on TL following exposure to ionizing radiation are currently very limited and focus on exposure to ionizing radiation in the general population, medical workers, and patients receiving radiotherapy treatment. The elevated level of natural chronic background radiation in Kerala coast (India) did not show any significant effect on TL of local residents [60]. No significant difference in TL was found between the inhabitants of high background radiation areas of Ramsar (Iran) and a control group from normal background radiation areas [61]. The leukocyte TL was shown to be significantly reduced in long-term radiation exposed personnel of the cardiac catheterization laboratory [62]. However, Tričković et al. [63] found no differences between the TL of hospital workers occupationally exposed to X-rays and the control group. Studies of Maeda et al. [64] and M’kacher et al. [65] demonstrated significant telomere shortening in patients receiving radiation therapy. A significant TL decrease has been demonstrated in peripheral blood lymphocytes of irradiated Chernobyl clean-up workers compared to the control group [66]. Taken together, the results reported here establish the critical importance of telomeres in the radiation response. 

Radiobiological studies of accelerated electrons are usually aimed at comparing the responses of healthy and diseased cells, as well as the effects of accelerators and conventional sources of ionizing radiation used in medicine. We have obtained results of the effects of accelerated electrons on the length of telomeres in normal blood and leukemic cells. However, a comparison of the effects of a laser-generated electron beam with a standard source of ionizing radiation is planned in upcoming projects. We consider the small sample of just one studied cell line to be another limitation of our study, which we intend to increase in the future. In addition, in our study, we implemented the Q-FISH method for evaluation of TLs, which does not allow the analysis of the actual length of telomeres in base pairs.

Here, we focused on the effect of radiation on telomeres, as telomeres are considered as targets of anti-tumor therapy. Telomere shortening with age protects against the uncontrolled proliferation and malignant transformation. In turn, the telomere maintenance via the activation of telomerase or alternative-lengthening of telomere is critical for the proliferation of malignant cells [67,68]. It is from this point of view that telomeres are considered as targets for medicinal compounds that are capable of chemically interacting with them. Radiation versus chemicals has a non-targeted, free radical-mediated effect on telomeres, which however also leads to their shortening. It is expected that any telomere maintenance mechanisms could be influenced by laser-generated electron beams, since radiation can perturb any cellular components [69]. Loss of telomere function is associated with genetic instability, cell cycle arrest, and apoptosis. Dysfunctional telomeres can limit tumorigenesis by activating p53-dependent cellular senescence and apoptosis [70]. One example of the effectiveness of telomere shortening to achieve an antitumor effect is the action of CML drug imatinib-mesylate (Gleevec), whose ability to shorten telomeres was shown in K562 cells [71]. Inhibition of K562 cell growth and proliferation by telomere erosion supports the potential therapeutic efficacy of laser-generated electron-induced telomere shortening demonstrated in our study.

## 4. Materials and Methods

### 4.1. Blood and K562 Cells Cultivation and Irradiation

Heparinized blood samples were collected by venipuncture from four healthy nonsmoking donors (two female and two male) of the same age group (30–32 years old) to avoid age differences in TL. 

The study protocol was approved by the Ethics Committee of the Institute of Molecular Biology of the National Academy of Sciences of the Republic of Armenia (IRB/IEC: IRB00004079, IORG 0003427, approval code # 07/2022, date of approval 30 September 2022). Informed consent was obtained from all study donors.

Electron beam irradiation of blood or K562 (human chronic myeloid leukemia) cells was carried out using laser-driven radiofrequency gun-based linear AREAL accelerator (CANDLE, Synchrotron Research Institute, Armenia), whose applications in life and materials sciences began in 2017 [9]. For irradiation, vacutainers (BD, Franklin Lakes, NJ, USA) with blood or K562 cells were placed in the sample holder facing vertically towards the beam coming from the direction of the vacuum window. 

Blood or K562 cells were irradiated on ice at doses of 0.5, 1.5, and 3.0 Gy with a dose rate of 2 Gy/min, and the beam charge was 10 pC, where the energy of electrons was 3 MeV, the pulse duration was 0.42 fs, and the pulse repetition rate was 2 Hz. Radiation doses were selected based on our previous findings, which showed that irradiation at the AREAL accelerator causes DNA damage at doses of 0, 2, 4, and 8 Gy in K562 cells [13] and CNVs at doses of 0.5, 1.5, and 3.0 Gy in blood cells [12]. Non-irradiated cell samples were prepared under the same conditions as irradiated cells and were used as controls.

After irradiation, blood cells were cultivated in RPMI-1640 (Gibco, Paisley, Scotland, UK), supplemented with 10% fetal bovine serum (Biochrom, Cambridge, UK), 100 IU/mL penicillin, and 0.1 mg/mL streptomycin with the addition of 10 μg/mL phytohemagglutinin-L (Santa Cruz Biotechnology, Dallas, TX, USA) at 37 °C for 72 h. K562 cells were maintained under the same conditions but without phytohemagglutinin-L. 

Metaphase chromosomes were prepared as previously described [72]. Blood cells were arrested in metaphase with 0.1 mg/mL colcemid (Santa Cruz Biotechnology, Dallas, TX, USA) 2 h before harvesting, then subjected to hypotonic shock with 0.075 M KCl (Santa Cruz Biotechnology, Dallas, TX, USA) for 15 min at 37 °C, followed by fixation with a 3:1 (*v*/*v*) mixture of methanol and acetic acid (Merck, Darmstadt, Germany). 

Interphase blood cells were analyzed on the same preparations as metaphase chromosomes. Interphase K562 cells were obtained using the same procedure, but the colcemid treatment was omitted.

### 4.2. Telomere Q-FISH

For detection of telomeric regions, FISH with telomeric probes (Telomere PNA FISH/Cy3; DAKO, Glostrup, Denmark) was carried out on blood and K562 cells according to the manufacturer’s recommendations, with minor modifications. The method of TL measurement based on the use of a PNA probe and appropriate digital image software (Telometer (freely available at https://demarzolab.pathology.jhmi.edu/telometer/ (accessed on 15 January 2023)) for the capture and quantification of fluorescence signals was described in Barbaro et al. [22].

The slides were dehydrated with ethanol (Merck, Darmstadt, Germany) series (70, 95, and 100%) for 2 min each, incubated in Tris-buffered saline (TBS) buffer (DAKO, Glostrup, Denmark), fixed in 3.7% formaldehyde (Merck, Darmstadt, Germany), and washed in TBS. Then, slides were immersed in pre-treatment solution (Telomere PNA FISH/Cy3; DAKO, Glostrup, Denmark) for 10 min, washed in TBS, and treated with pepsin (Merck, Darmstadt, Germany) solution for 5 min at 37 °C. After washing in TBS, the preparations were dehydrated with ethanol series (70, 95, and 100%). In total, 20 µL of hybridization mixture containing telomere PNA Probe/Cy3 was added to the slide, and DNA was denatured by heat for 5 min at 87 °C and hybridized for 2 h in a dark chamber. After hybridization, the preparations were rinsed in PBS (Merck, Darmstadt, Germany), washed in a wash solution for 5 min at 65 °C, and treated with 70% formamide (Merck, Darmstadt, Germany) for 5 min. Then, the preparations were washed twice with TBS and dehydrated in an ethanol series (70, 95, and 100%). The preparations were stained with DAPI-containing VECTASHIELD Antifade (DAKO, Wiesbaden, Germany). Fluorescence signals on metaphase chromosomes and interphase nuclei were visualized under a Zeiss fluorescent microscope with the MetaSystems imaging system (GmbH, Altlussheim, Germany) using a 100× objective. 

According to Vera and Blasco [19], 15–20 metaphases and 30 interphases are sufficient to obtain reliable TL measurements by Q-FISH. Cabuy et al. [73] were able to identify subpopulations of ɣ-rays irradiated mouse and human cells with differing TLs by metaphase and interphase Q-FISH when analyzing 15 cells. In our study, 30–40 metaphases and interphases in three replicates were captured in normal blood cells from each of four donors for each dose of irradiation and in the control, a total of 120–160 cells. Accordingly, 120–160 interphases were analyzed in K562 cells in four replicates for each dose of irradiation and in the control.

Total telomere fluorescence intensity per cell was determined with ImageJ (https://imagej.nih.gov/ij/ (accessed on 15 January 2023)) software (version: IJ1.46r) plugin Telometer (version: 3.1.0) and expressed in arbitrary units (a.u.) [22]. The total fluorescence intensity of each cell is calculated as the sum-of intensity of all the pixels that belong to the telomeres recorded in the cell. Differences in TL were determined as a function of fluorescence intensity. Short telomeres were defined as a TL below the 20th percentile of the corresponding control.

### 4.3. Statistical Analysis

For statistical analysis, STATGRAPHICS Centurion 16.2 (Stat-Point Technologies, Inc.; Warrenton, VA, USA) and SPSS version 19 (SPSS, Inc., an IBM Company, Chicago, IL, USA) were used. The ANOVA test was performed for comparisons between four donors. The Kruskal–Wallis test was used for multiple comparisons of the Q-FISH results. The Kolmogorov–Smirnov test was applied for the distribution analysis. Correlations between the radiation dose and TL were calculated using Spearman’s rank correlation analysis. A *p* value less than 0.05 was considered statistically significant.

## 5. Conclusions

Strategies targeting telomere maintenance offer significant opportunity for the development of effective treatments. Here, we report on the validation of a telomere Q-FISH assay in interphases, which provides similar quality data on TL dynamics as conventional Q-FISH analysis of metaphase spreads in normal blood cells irradiated with laser-driven electron beams. Based on this initial study, we conclude that irradiation with laser-generated electron beams induced dose-dependent telomere shortening. Therefore, TL can be considered a sensitive marker of radiation effect. In turn, telomere shortening, leading to chromosomal aberrations, genomic instability, and cell death, is considered as one of the indicators of the expected antitumor effect. We also found that K562 leukemic cells are more sensitive to increasing doses of radiation, which opens up the possibility of adjusting beam parameters to increase the radiosensitivity of tumor cells compared to normal cells. The results may contribute to the further development of telomere-based radiotherapy approaches with the aim of minimizing radiation side effects.

A better understanding of the effects of laser-generated electron beams may be achieved through further studies of the interactions between telomere maintenance mechanisms with oxidative stress and DNA damage repair pathways in a wider range of cell lines and in vivo models, compared with the effects of traditional sources of ionizing radiation.

## Figures and Tables

**Table 1:** TLs in arbitrary units (a.u.) in metaphase chromosomes (n = 30–40) and interphase nuclei (n = 30–40) of normal blood leukocytes of four donors (D1, D2, D3, and D4).

Irradiation Variant, Gy	TLs in Metaphases (mean ± SE), a.u.	TLs in Interphases (mean ± SE), a.u.
Control (D1)	736.82 ± 18.54	587.38 ± 14.01
0.5 (D1)	658.76 ± 15.45	527.45 ± 18.44
1.5 (D1)	553.20 ± 19.67	436.78 ± 19.50
3.0 (D1)	548.58 ± 12.04	421.81 ± 11.42
Control (D2)	767.77 ± 15.89	584.60 ± 16.48
0.5 (D2)	652.52 ± 22.09	516.52 ± 12.32
1.5 (D2)	578.64 ± 20.83	461.57 ± 16.88
3.0 (D2)	562.05 ± 19.71	426.35 ± 13.82
Control (D3)	776.05 ± 30.06	586.98 ± 30.05
0.5 (D3)	651.62 ± 28.33	513.79 ± 16.97
1.5 (D3)	551.78 ± 25.95	451.69 ± 16.50
3.0 (D3)	546.88 ± 21.08	453.44 ± 25.28
Control (D4)	734.80 ± 28.02	587.22 ± 19.11
0.5 (D4)	630.18 ± 32.54	522.62 ± 19.93
1.5 (D4)	563.15 ± 28.15	461.60 ± 24.58
3.0 (D4)	555.34 ± 26.40	432.20 ± 19.81

**Table 2:** The percentage of telomeres, shorter than the 20th percentile of the control variant in blood and K562 cells irradiated with different doses of laser-generated electron beam. a.u.—arbitrary units.

Cells (20th Percentile of Control)	0.5 Gy (%)	1.5 Gy (%)	3.0 Gy (%)
Blood metaphases (640.40 a.u.)	47.50	79.17	81.67
Blood interphases (462.48 a.u.)	22.70	55.97	63.41
K562 interphases (363.01 a.u.)	23.17	55.49	79.88

## Data Availability

Dataset available on request from the authors.
